# Triglyceride profiling in adipose tissues from obese insulin sensitive, insulin resistant and type 2 diabetes mellitus individuals

**DOI:** 10.1186/s12967-018-1548-x

**Published:** 2018-06-26

**Authors:** Haya Al-Sulaiti, Ilhame Diboun, Sameem Banu, Mohamed Al-Emadi, Parvaneh Amani, Thomas M. Harvey, Alex S. Dömling, Aishah Latiff, Mohamed A. Elrayess

**Affiliations:** 1 Toxicology and Multipurpose Lab, Anti Doping Laboratory Qatar, Sports City, Doha, Qatar; 2 Department of Economics, Mathematics and Statistics, Birkbeck, University of London, London, WC1E 7HX UK; 3 General Surgery Department, Al-Emdi Hospital, Doha, Qatar; 4 Department of Drug Design, University of Groningen, A. Deusinglaan 1, 9713 AV Groningen, Netherlands; 5 Division of Medicine, Royal Free and University College Medical School, University College London, London, UK

**Keywords:** Lipidomics, Adipose tissue, Triaclyglycerols, Insulin sensitivity, Insulin resistance, Type 2 diabetes mellitus

## Abstract

**Background:**

Lipid intermediates produced during triacylglycerols (TAGs) synthesis and lipolysis in adipocytes interfere with the intracellular insulin signaling pathway and development of insulin resistance. This study aims to compare TAG species and their fatty acid composition in adipose tissues from insulin sensitive (IS), insulin resistant (IR) and type 2 diabetes mellitus (T2DM) obese individuals.

**Methods:**

Human subcutaneous and omental adipose tissue biopsies were obtained from 64 clinically characterized obese individuals during weight reduction surgery. TAGs were extracted from the adipose tissues using the Bligh and Dyer method, then were subjected to non-aqueous reverse phase ultra-high performance liquid chromatography and full scan mass spectrometry acquisition and data dependent MS/MS on LTQ dual cell linear ion trap. TAGs and their fatty acid contents were identified and compared between IS, IR and T2DM individuals and their levels were correlated with metabolic traits of participants and the adipogenic potential of preadipocyte cultures established from their adipose tissues.

**Results:**

Data revealed 76 unique TAG species in adipose tissues identified based on their exact mass. Analysis of TAG levels revealed a number of TAGs that were significantly altered with disease progression including C46:4, C48:5, C48:4, C38:1, C50:3, C40:2, C56:3, C56:4, C56:7 and C58:7. Enrichment analysis revealed C12:0 fatty acid to be associated with TAGs least abundant in T2DM whereas C18:3 was found in both depleted and enriched TAGs in T2DM. Significant correlations of various adipose tissue-derived TAG species and metabolic traits were observed, including age and body mass index, systemic total cholesterol, TAGs, and interleukin-6 in addition to adipogenic potential of preadipocytes derived from the same adipose tissues.

**Conclusion:**

Pilot data suggest that adipose tissues from obese IR and T2DM individuals exhibit TAG-specific signatures that may contribute to their increased risk compared to their IS counterparts. Future experiments are warranted to investigate the functional relevance of these specific lipidomic profiles.

**Electronic supplementary material:**

The online version of this article (10.1186/s12967-018-1548-x) contains supplementary material, which is available to authorized users.

## Background

Adipose tissue is the main site for storing and mobilizing energy in response to metabolic demand. Obesity is associated with changes in the structure and function of the adipose tissue, leading to progression of insulin resistance and type 2 diabetes mellitus (T2DM) [1]. However, a subset of obese individuals, known as the insulin sensitive (IS) obese, maintain insulin sensitivity and exhibit better adipose tissue functions compared to equally obese insulin resistant (IR) counterparts [2]. Obesity triggers hypertrophy of adipocytes within the subcutaneous (SC) adipose tissues to enable accumulation of excess triacylglycerols (TAGs). Additional energy intake causes further fat accumulation within the omental (OM) depot, which is associated with ectopic fat deposition in the liver, skeletal muscle and heart tissues [3]. The subsequent hyperinsulinemia inhibits hormone sensitive lipase and triggers the lipoprotein lipase causing additional glucose intolerance, hyperinsulinemia, hypertriglyceridemia and higher risk of insulin resistance in these tissues [4].

Analysis of complex biological systems has become possible by the newly emerging metabolomics techniques where metabolites serve as direct indicators of biochemical activity of complex phenotypes such as insulin resistance and T2DM [5]. In this context, lipidomics studies were utilized to study differences between SC and OM depots. These studies have revealed depot-specific enrichment of specific TAGs, glycerophospholipids, and sphingolipids and differences in the association of lipid species with body mass index, inflammation and insulin sensitivity [6, 7]. Although TAGs themselves are unlikely to be signaling molecules, an increasing body of evidence suggests that lipid intermediates produced during TAG synthesis or breakdown interfere with the intracellular insulin signaling pathway and contribute to the development of insulin resistance, including free fatty acids, diacylglycerols and ceramides [8]. Indeed, elevated fatty acid efflux from the adipose tissue stimulates TAG synthesis in the liver and triggers stress of endoplasmic reticulum and stimulation of June kinase pathway in the adipose tissues [9, 10]. This leads to an overload of TAG’s synthetic capacity, causing an increase in both diacylglycerols (DAGs) and ceramide levels and further development of insulin resistance in adipocytes [11].

Despite various studies investigating lipidomic differences in human serum and adipose tissues in relation to insulin sensitivity, no studies have compared differences in TAG signatures and their fatty acid composition in adipose tissues from IS, IR and T2DM obese individuals and their correlations with mediators of metabolic disease. Identification of the fatty acids that are enriched or depleted in tissues from insulin resistance and T2DM individuals could shed light on their functional role in disease progression, thus providing potential novel targets for therapeutic intervention. The aims of this study were to profile TAG species and measure their levels in two fat depots and to compare their fatty acid composition between IS, IR and T2DM individuals.

## Methods

### Materials

Interleukin 6 (IL-6) and leptin ELISAs were from R&D systems (Abingdon, UK). Insulin ELISA was from Mercodia Diagnostics (Uppsala, Sweden). 4′,6-Diamidino-2-phenylindole (DAPI), and LipidTOX Green Neutral Lipid were from Life Technologies (Warrington, UK). Other chemicals and reagents were from Sigma (Munich, Germany).

### Cohort

Participants’ recruitment criteria were described previously [12]. Briefly, 64 consented obese individuals undergoing bariatric surgery at AlEmadi hosptial (Doha, Qatar) were recruited. Protocols were approved by Institutional Review Board of ADLQ (X2017000224). Blood was taken prior to operation and 1–5 g of abdominal SC and OM adipose tissues biopsies were collected during the surgery and stored at − 80 °C until use. Plasma cholesterol, fasting glucose and liver function enzymes were measured by COBAS INTEGRA (Roche Diagnostics, Basil). IL-6, leptin and insulin were determined using commercially available ELISA. Insulin resistance was computed by homeostatic model assessment (HOMA-IR) [13] using 30th percentile (HOMAIR = 2.4) as a threshold point. Accordingly, subjects were dichotomized into IS (HOMA-IR < 2.4, n = 18, 3 males and 15 females) and IR (HOMA-IR > 2.4, n = 35, 9 males and 26 females). Eleven participants were clinically diagnosed with T2DM (8 males and 3 females).

### Preadipocyte culture and differentiation

Stromal vascular fraction (SVF) cells were obtained by collagenase digestion of adipose tissues as described previously [12]. Cell pellets were re-suspended in stromal medium containing Dulbecco’s modified Eagle’s Medium-F12 (DMEM-F12) supplemented with 10% fetal bovine serum (FBS) and Penicillin/Streptomycin, then maintained at 37 °C with 5% CO_2_ until confluence. To induce differentiation, early passaged stromal vascular fraction (SVF)-derived preadipocytes (passages 1–3) were grown at 2 × 10^4^/cm^2^ in stromal medium overnight, then incubated in differentiation medium (DMEM-F12, 3% FBS, 33 μM biotin, 17 μM d-pantothenate, 1 μM dexamethasone, 250 μM of methylisobutylxanthine, 0.1 μM human insulin, 5 μM of Peroxisome proliferator-activated receptor gamma PPARγ agonist, rosiglitazone) for 7 days, followed by 12 days in maintenance medium containing the same components as the differentiation medium but omitting methylisobutylxanthine and rosiglitazone. Differentiation potential (adipogenic capacity) was determined as a percentage of lipidtox positive stained cells to total number of stained nuclei (DAPI).

### Sample preparation

Human SC and OM adipose tissue specimens from IS, IR and T2DM individuals were extracted using the Bligh and Dyer Method [14]. Homogenization of tissue was carried out in the gentle MACS Dissociator (Miltenyi Biotech, Germany) with one volume of PBS for every gram of tissue. Following tissue homogenization, 1 mL of each sample solution was transferred into a separate 15 mL Falcon tube, and 3 mL of 3:1 ratio of Chloroform:MeOH were added into each tube. One microliter of PBS was added and samples were centrifuged at 3000 RPM for 20 min at room temperature. The organic layer (bottom layer) was carefully transferred into new 15 mL Falcon tubes and evaporated to dryness under a stream of high purity nitrogen. Samples were then reconstituted with 1:1:1 mixture of hexane, isopropanol, acetonitrile. Subsequently the extracts were analysed using data dependent full scan MS and MS/MS acquisition using the Thermo LTQ VelosPro dual cell linear ion trap mass spectrometer (Thermo Fisher Scientific, San Jose, CA, USA).

### Sample analysis

Separation of TAGs was carried out using non-aqueous reverse phase UHPLC separation (NARP), on a Dionex Ultimate 3000 UHPLC system, using acetonitrile w/0.1% formic acid (eluent A), and isopropanol w/10 mM ammonium formate (eluent B) as the mobile phase. The column was a Phenomenex UHPLC C30 core shell, 150 mm × 2.1 mm and 2.7 µm particle size (Phenomenex Torrance CA, USA). Gradient conditions started with 5% B held for 2 min, then raised to 50% B at 30 min, held for 10 min and then reduced to 5% B at 45 min and held for further 5 min.

### Mass spectrometry

MS analyses were conducted using the Thermo LTQ VelosPro dual cell linear ion trap mass spectrometer (Thermo Fisher Scientific, San Jose, CA, USA), acquiring both full scan MS and subsequent data dependent full scan MS/MS product ion spectra with wide band activation. Target parent ions were automatically selected from an inclusion list. The low resolution full scan analysis provides molecular parent masses (M^+^NH_4_^+^). These parent ion full scan MS/MS analysis provided further elucidation of possible structures represented in each lipid (fatty acid composition). Relative abundances of each identified TAG were estimated from the height values for each extracted ion current profile for parent masses of each compound (M^+^NH_4_^+^).

### Separation by equivalent carbon number

The above UHPLC conditions (NARP) provide separations of TAGs by their equivalent carbon number (ECN). The ECN is calculated, from the total number of non-glycerol carbons in the TAG minus twice the number of the double bonds in the molecule (ECN = CN − 2DB). NARP eluted the TAGs from lower to higher ECN with increasing percent B in the eluent. NARP-HPLC is commonly used for TAG separation because it works on both the chain and absolute height or area counts for each identified TAG. As some of the TAG may not show baseline resolution, the height counts were chosen to better represent the TAG.

### Statistical analysis

All statistical analyses were carried out using R version 3.2.1 and SIMCA 13.0.1 software (Umetrics, Sweden). Variables with skewed distributions were log transformed or taken the square root of as appropriate to ensure normality. An initial PCA was conducted to identify components that explain large proportion of the TAG variance. A repeated measures linear model incorporating confounders: gender, age, BMI, PC1 and PC2 (derived from earlier principle component analysis, PCA) and covariates: tissue and diabetic status (IS, IR, T2DM) was used to assess the differences in each TAG between the two tissues and amongst the insulin/diabetes groups. The model was based on repeated measures statistics since a TAG measurement from an individual was taken from two separate tissues: SC and OM. The model allows the individual inherent variation to be taken out of the total variance. Such enhanced modelling of the error structure increases the model’s ability to detect significance of covariate effects. Nonetheless, we have repeated the analysis using the standard linear model and confirmed the superiority of the repeated measures linear model counterpart. The linear model was sometimes used when fitting the repeated measures model was not possible due to missing data. False discovery rate (FDR) multiple testing correction was also performed on the differentially expressed TAG species identified between adipose tissues from IS, IR and T2DM individuals. Fatty acid enrichment amongst diabetes/tissue significant TAGs was assessed using the one tailed Wilcoxon sum of the ranks test on the list of metabolites that differed significantly between IS, IR and T2DM after correcting for covariates including gender, age, BMI, PC1 and PC2. The analysis was based on assessing the likelihood of randomly observing a given fatty acid that often amongst highly ranked TAGs along the list of all TAGs ordered by p value as follows: For each of the following contrasts: subcutaneous versus omental, IR versus IS, IR versus T2DM and IS versus T2DM, TAGs were ranked by their p values and a given fatty acid mapped to the ranks of TAGs within which it is found. The analysis proceeds by assessing the likelihood of obtaining the observed sum of fatty acid identified ranks by chance. If the fatty acid is observed amongst the significant TAG at the top of the list, the sum of the ranks would be too small to be explained by chance alone; hence the null hypothesis is rejected in favor of enrichment. Enrichment hits failed to remain significant after FDR multiple testing correction but data was reported because of agreement with literature as elaborated in “Discussion” section. A similar test was used to assess enrichment in constituent fatty acid saturation levels.

## Results

### General characteristics of participants

Sixty-four (44 females and 20 males) obese and morbidly obese (BMI = 43.1 ± 7.5 kg/m^2^) participants were recruited from amongst patients undergoing weight reduction surgery. Participants exhibited hyperleptinemia and hyperinsulinemia and were dichotomized into IS and IR groups based on their HOMA-IR index and into T2DM based on their medical records. Compared to BMI-matched IS and IR subjects, T2DM individuals were older and had higher circulating levels of TAG and lower leptin (Table 1). Compared to females, males had higher mean arterial blood pressure (MAP) (93.7 vs 84.7, p > 0.01) and lower HDL (1.1 vs 1.5, p = 0.05) and leptin (42.0 vs 67.3, p > 0.01) (Additional file 1: Table S1). IS males had lower HOMA-IR than their age and BMI-match IS females, whereas IR males had higher HOMA-IR than their age, but not BMI, matched females (Additional file 1: Table S1). Compared to obese subjects (n = 26), the morbidly obese participants (n = 46) had significantly higher BMI, SBP, IL-6, FPG and HOMA-IR (Additional file 1: Table S2).

**Table 1:** General characteristics of participants

Variables	IS	IR	T2DM	P value				IS+IR	P value
	(N = 18)	(N = 35)	(N = 11)	ANOVA	IS vs IR	IS vs T2DM	IR vs T2DM	(N = 46)	IS+IR vs T2DM
Age (years)	32.09 (9.7)	30.26 (9.3)	43.57 (9.4)	0.000	0.739	0.017	0.003	30.9 (9.4)	0.001
BMI (kg m^−2^)	41.44 (7.0)	43.31 (6.9)	45.53 (9.6)	0.38	0.583	0.394	0.731	42.7 (6.9)	0.322
SBP (mmHg)	119.0 (13.8)	122.67 (15.7)	132.14 (11.6)	0.13	0.616	0.111	0.268	121.5 (15.1)	0.075
DBP (mmHg)	66.1 (9.8)	69.98 (12.7)	73.33 (7.0)	0.3	0.422	0.369	0.783	68.7 (11.9)	0.35
MAP	83.4 (8.1)	88.08 (12.4)	93.53 (8.9)	0.11	0.288	0.130	0.499	86.6 (11.3)	0.15
Cholesterol (mmol/L)	4.4 (0.9)	4.6 (1.2)	5.22 (0.9)	0.24	0.785	0.215	0.364	4.5 (1.1)	0.122
LDL (mmol/L)	2.73 (0.8)	2.91 (0.9)	3.1 (1.0)	0.55	0.693	0.593	0.855	2.8 (0.8)	0.466
HDL (mmol/L)	1.33 (0.4)	1.46 (0.9)	1.29 (0.3)	0.74	0.793	0.990	0.844	1.4 (0.8)	0.678
Triglyceride (mmol/L)	1.13 (0.6)	1.27 (0.7)	1.91 (1.1)	0.04	0.729	0.031	0.072	1.2 (0.6)	0.015
Leptin (ng/mL)	64.36 (25.5)	60.7 (21.9)	39.16 (31.5)	0.06	0.854	0.055	0.091	62.1 (23.1)	0.021
Adiponetin (ng/mL)	3.24 (2.2)	3.62 (1.9)	3.47 (2.4)	0.88	0.866	0.982	0.992	3.5 (1.9)	0.979
IL-6 (pg/mL)	3.28 (1.8)	3.72 (1.8)	4.03 (2.2)	0.58	0.683	0.627	0.913	3.6 (1.8)	0.521
FBG (mmol/L)	5.73 (2.5)	12.76 (8.4)	12.84 (6.5)	0.000	0.001	0.072	1.000	10.3 (7.7)	0.439
Insulin (mIU/L)	6.33 (1.9)	12.6 (10.0)	11.92 (6.4)	0.01	0.010	0.241	0.976	10.3 (8.6)	0.633
HOMA-IR	1.56 (0.6)	4.86 (2.0)	6.6 (3.2)	0.000	0.000	0.000	0.080	3.7 (2.3)	0.005

*BMI* body mass index, *SBP* systolic blood pressure, *DBP* diastolic blood pressure, *MAP* mean arterial blood pressure, *LDL* low density lipoprotein, *HDL* high density lipoprotein, *IL-6* interleukin 6, *FPG* fasting blood glucose, *HOMA-IR* homeostatic model assessment of insulin resistance. Data are presented as mean (SD). Differences between IS, IR and T2DM were tested by ANOVA. Differences between (IS+IR vs T2DM) were tested by the independent-sample t test or Mann–Whitney U test. A p value significance level of 0.05 was used

### Differences in TAG content between omental and subcutaneous adipose tissues

Using a non-targeted approach, a comprehensive parent mass list of 120 identified TAGs was created, of which 76 TAG species were identified (Appendix, Table 6) based on their molecular weights and peak heights. A linear model was used to assess depot-specific TAG associations after correcting for participant diabetes group, gender, PC1 and PC2 (refer to “methods” section). Analysis revealed 7 TAGs that were significantly different between SC and OM tissues. C53:5, C51:3, C50:4, C59:1, C54:6 and C50:2 were higher in OM than SC. C38:1 was higher in the SC compared to OM tissues. The full scan MS/MS analysis revealed the fatty acid composition for each identified TAG (Table 2).

**Table 2:** Differential TAG species identified between subcutaneous and omental adipose tissues

ID	TAG	MW	Fatty acid composition	Fatty acids identities	Fold change (SC-OM)	P value
TAG47	C53:5	866.7	C17:0, C17:1, C19:4	Heptadecanoic acid, *cis*-10-heptadecanoic acid, C19:4	0.17	0.01
TAG2A	C38:1	664.7	C18:1, C16:0, C4:0	Oleic acid, palmitic acid, butyric acid	− 0.44	0.02
TAG36	C51:3	842.6	C18:1, C16:1, C17:1	Oleic acid, palmitoleic acid, *cis*-10-heptadecenoic acid	0.32	0.03
TAG31	C50:4	826.7	C18:2, C18:2, C14:0	Linoleic acid, linoleic acid, myristic acid	0.37	0.03
TAG81	C59:1	958.8	C23:0, C18:0, C18:1	Tricosanoic acid, stearic acid, oleic acid	0.16	0.04
TAG53A	C54:6	878.7	C18:2, C18:2, C18:2	Linoleic acid, linoleic acid, linoleic acid	0.17	0.04
TAG33	C50:2	830.8	C18:2, C16:0, C16:0	Linoleic acid, palmitic acid, palmitic acid	0.19	0.05

Molecular weight (MW), fatty acid composition, fatty acid identity, fold change in SC tissue compared to OM are also indicated

### TAGs with varying levels between IS, IR and T2DM

A linear model was used to assess TAG associations with participant groups after correcting for possible confounders (refer to “Methods” section). A number of TAGs were significantly decreased in T2DM compared to IS and/or IR including C46:4, C48:5, C48:4, C38:1, C50:3 and C40:2 whereas a number of TAGs were increased in T2DM compared to the other two groups including C56:3, C56:4, C56:7 and C58:7. No significant differences in TAGs between IS and IR groups was detected. Table 3 summarizes the list of differentially expressed TAGs with their fatty acids compositions. When looking at gender versus group (IS, IR and T2DM) interaction, there were no FDR significant interaction effects. However, when considering BMI versus group interaction, two TAG species showed FDR significant interaction effects including C40:2 and C53:4. Whereas the former (C40:1) shows more pronounced decrease in T2DM compared to IS in low BMI than in high BMI, the latter (C53:4) shows a more pronounced increase in low BMI than in high BMI (Additional file 1: Table S3).

**Table 3:** Differentially expressed TAG species identified between adipose tissues from IS, IR and T2DM individuals

ID	TAG	MW	Fatty acid composition	Fatty acids identities	Comparison	Fold change	FDR p value
TAG16A	C46:4	770.7	C18:2, C18:2, C10:0	Linoleic acid, linoleic acid, capric acid	IS vs T2DM	− 0.62	0.005
					IR vs T2DM	− 0.53	0.01
TAG21	C48:5	796.7	C18:2, C18:3, C12:0	Linoleic acid, linolenic acid, lauric acid	IR vs T2DM	− 0.39	0.0005
					IS vs T2DM	− 0.38	0.0013
TAG22	C48:4	798.7	C18:2, C18:2, C12:0	Linoleic acid, linoleic acid, lauric acid	IR vs T2DM	− 0.96	0.002
TAG2A	C38:1	664.7	C18:1, C16:0, C4:0	Oleic acid, palmitic acid, butyric acid	IS vs T2DM	− 1.00	0.0007
TAG32	C50:3	828.8	C16:1, C16:1, C18:1	Palmitoleic acid, palmitoleic acid, oleic acid	IR vs T2DM	− 0.78	1.37E–05
					IS vs T2DM	− 0.76	5.62E–05
TAG61	C56:7	904.8	C20:4, C18:1, C18:2	Arachidonic acid, oleic acid, linoleic acid	IS vs T2DM	0.81	0.0006
					IR vs T2DM	0.74	0.001
TAG64	C56:4	910.8	C18:1, C18:2, C20:1	Oleic acid, linoleic acid, gadoleic acid	IS and IR vs T2DM	0.98	0.004
TAG65	C56:3	912.8	C20:1, C18:1, C18:1	Gadoleic acid, oleic acid, oleic acid	IR vs T2DM	0.51	0.002
					IS vs T2DM	0.54	0.002
TAG7	C40:2	690.7	C6:0, C16:0, C18:2	Caproic acid, palmitic acid, linoleic acid	IS vs T2DM	− 1.07	1.26E–05
					IR vs T2DM	− 0.83	0.0002
TAG74	C58:7	934.8	C18:6, C24:0, C16:1	C18:6, lignoceric acid, palmitoleic acid	IS and IR vs T2DM	0.48	0.007
TAG75	C58:4	938.7	C18:3, C24:0, C16:1	Linolenic acid, lignoceric acid, palmitic acid	IR vs T2DM	0.69	0.0005
					IS vs T2DM	0.73	0.0005
TAG9	C42:2	718.7	C18:2, C12:0, C12:0	Linoleic acid, lauric acid, lauric acid	IS vs T2DM	− 0.77	0.0008
					IR vs T2DM	− 0.71	0.001

*MW* molecular weight, fatty acid composition, fatty acid identity, fold change between specified groups are also indicated

**Fig. 1:**
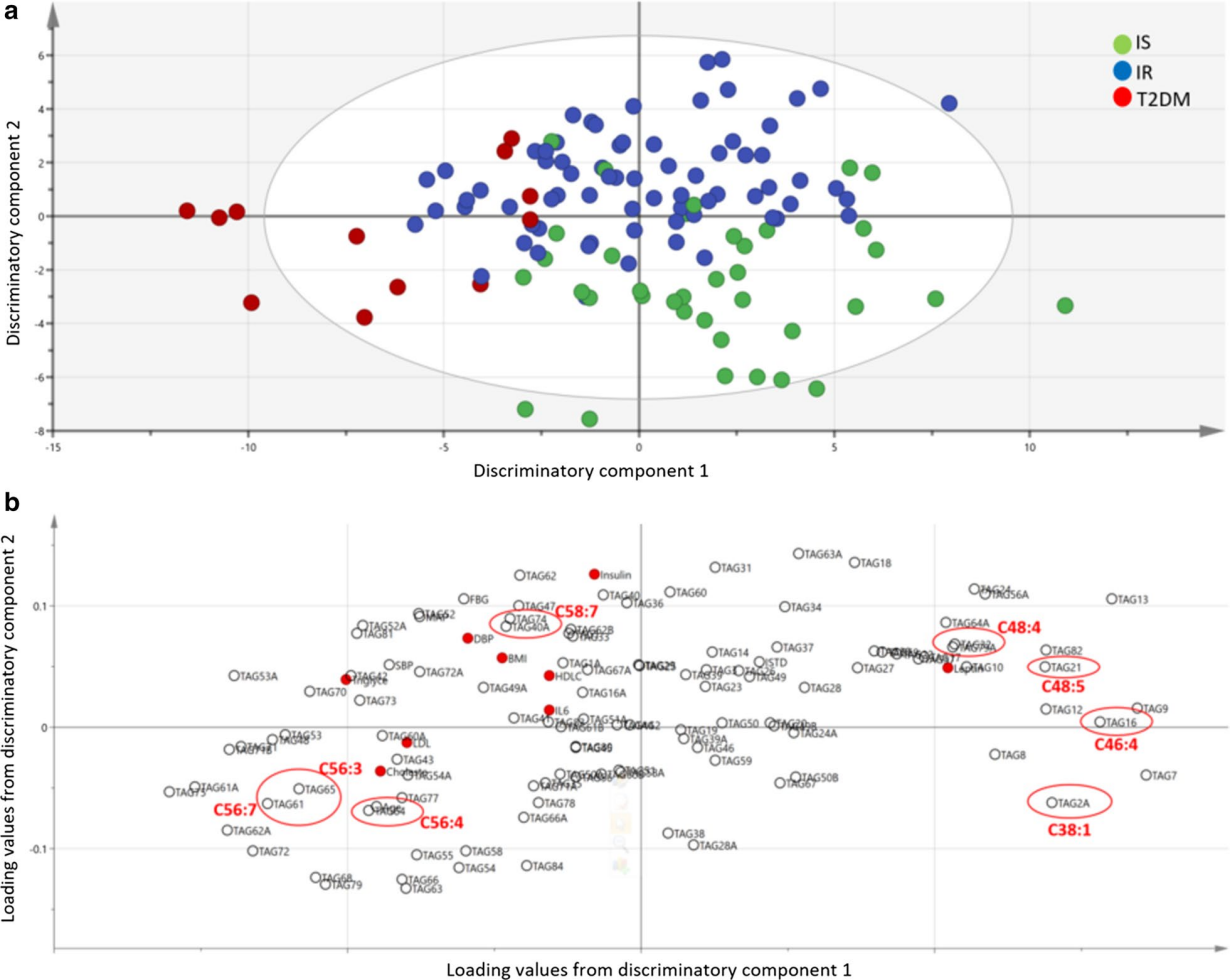
OPLS-DA model comparing adipose tissue-derived TAGs from IS, IR and T2DM individuals. **a** A score plot showing the class-discriminatory component 1 (*x*-axis) versus class-discriminatory component 2 (*y*-axis). **b** The corresponding loading plot showing similar TAG/diabetes group associations to the linear model (circled in red)

An orthogonal partial least square discriminate analysis (OPLS-DA) comparing subjects from IS, IR and T2DM revealed two significant class-discriminatory components (R2X = 0.18, R2Y = 1, R2Q2 = 0.27, CV-ANOVA p = 0.0001) (Fig. 1). The score plot in Fig. 1a indicates an x-axis differentiating the T2DM group from IS and IR; the latter two groups being rather separated along the y-axis. The corresponding loading score, shown in Fig. 1b, features similar TAG/group associations to those obtained with the linear model (shown in Table 3). Specifically, lower amounts of C38:1, C46:4, C48:5 and C48:4 as opposed to higher levels of C58:7, C56:4, C56:4 and C56:7 in the T2DM group (also circled in red, Fig. 1b).

**Fig. 2:**
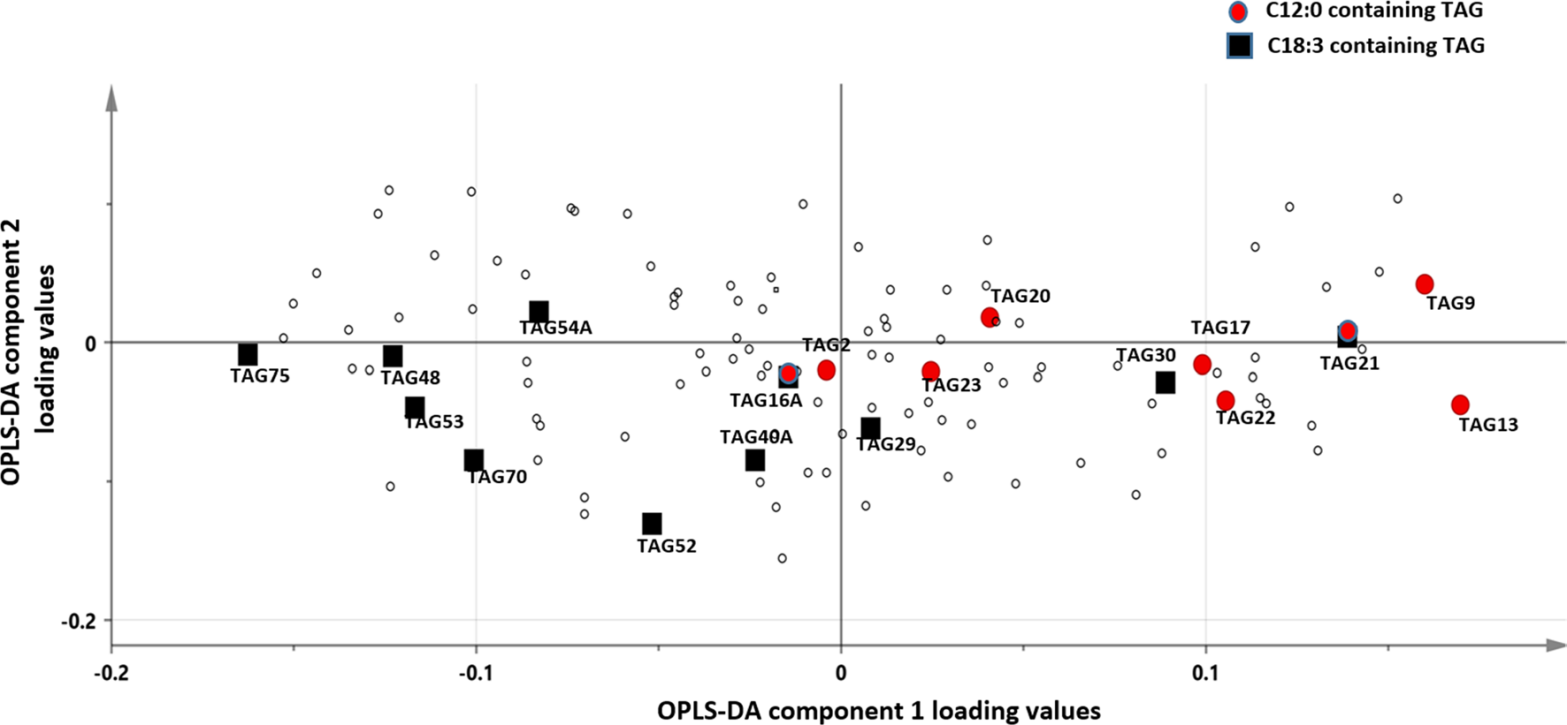
An OPLS-DA loading plot showing the spread of C12:0 and C18:3 containing TAG along the x-axis found previously (Fig. 1a) to differentiate T2DM from IS+IR subjects. Unlike the C12:0 containing TAGs, the TAGs comprising C18:3 feature on both sides of the x-axis implying depletion of certain recipient TAGs (right side) as oppose to enrichment of others (left side) with diabetes

**Fig. 3:**
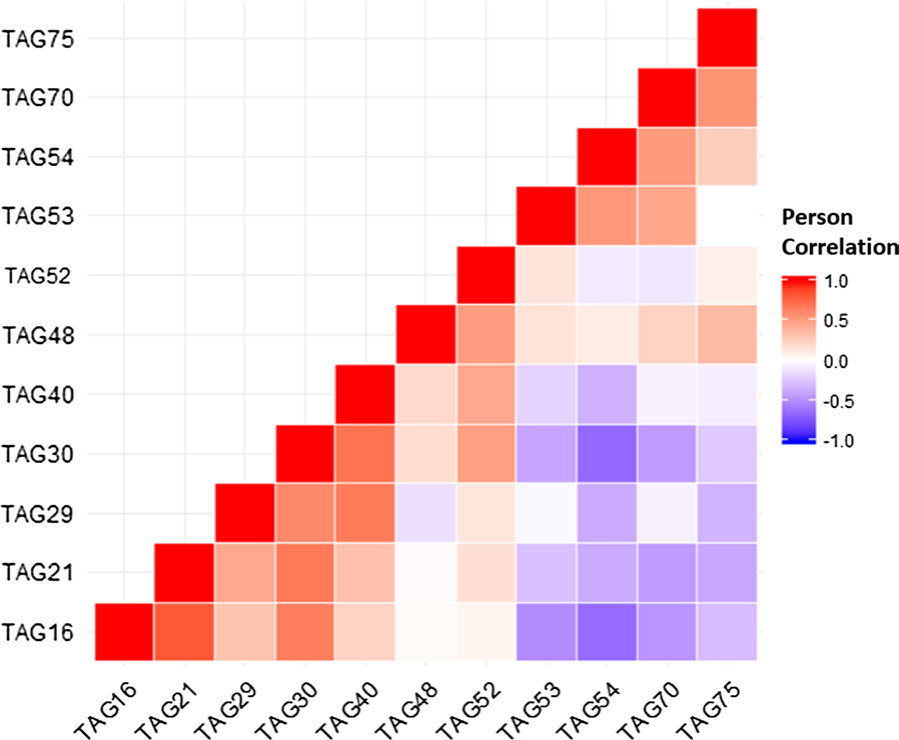
A triangular heatmap showing correlations between C18:3 carrying TAGs

In order to study the possible enrichment/depletion of certain fatty acid constituents of TAGs in association with insulin sensitivity or diabetes, an enrichment analysis based on the Wilcoxon sum of the ranks test was conducted (refer to “Methods” section). The results of the analysis are presented in Table 4 and further illustrated on Fig. 2. Overall, C12:0 appears to be associated with TAGs least abundant in T2DM in both tissues whereas C18:3 is found in both depleted and enriched TAGs in T2DM (both sides of the x-axis in Fig. 2). This could be justified by a potentially induced flow of C18:3 in certain recipient TAGs at the expense of other TAGs with diabetes. Further supporting this are the observed negative correlations between depleted and enriched C18:3 carrying TAGs (Fig. 3). Interestingly, many of the C12:0 and C18:3 containing TAGs, including TAG21, TAG22, TAG75 and TAG9, were previously identified as significantly changing in level with diabetes by the linear model (Table 3).

**Table 4:** TAG fatty acid association with tissue and diabetes/insulin sensitivity groups

Compared groups	Fixed variable	Fatty acid	p value
IR × T2DM	SC	C12:0	0.045
	SC	C18:3	0.048
	OM	C12:0	0.016
	(Full model) SC+OM	C12:0	0.025
IS × T2DM	OM	C12:0	0.03
	OM	C18:3	0.048
SC × OM	(Full model) IS+IR+T2DM	C18.3	0.027

Analysis conducted using the Wilcoxon sum of the ranks test indicates fatty acids that were overrepresented amongst hit TAGs when comparing the groups specified in column 1. Comparing IS, IR and T2DM was done in individual tissues as well as when pooling data from the two tissues. Similarly, tissues were compared per group and when groups were combined (column 2). Only significant results are shown at a nominal p value of 0.05

Furthermore, C18:3 was also found to have a strong tissue signature featuring frequently amongst highly ranked TAGs from statistical analysis of TAG association with tissue type (data not shown). However, C18:3 does not feature amongst the TAGs found to significantly differ between tissues by the linear model (Table 2). This indicates that the collective tissue changes in C18:3 containing TAGs were rather subtle.

The Wilcoxon sum of the ranks analysis was also used to look for enrichment of fatty acid saturation level amongst the highly ranked significant TAGs from comparison of tissue/diabetes-insulin sensitivity groups but no significance was detected.

### Correlation of TAG species with mediators of metabolic disease

A step-wise regression was performed to identify the best TAG predict of various traits including age, BMI, systemic TAG, total cholesterol, IL-6 and HOMA-IR, SC and OM adipogenic capacity reported previously [12, 15] and shown in Additional file 2: Figure S1. Table 5 lists TAG species identified with significant (p = 0.0001) association with various metabolic traits and shows their importance and fatty acid compositions.

**Table 5:** List of TAGs associated with metabolic traits such as age, BMI, TC, TG, IL-6, HOMA-IR, SC and OM adipogenic capacity

Metabolic trait	R^2^	Importance	TAG	MW	Fatty acid composition	Fatty acids identities
Age	0.4	0.12	C56:1	916.8	C20:0, C18:0, C18:1	Arachidic acid, stearic acid, oleic acid
		0.12	C54:8	874.8	C18:3, C18:3, C18:2	Linolenic acid, linolenic acid, linoleic acid
BMI	0.5	0.12	C57:1	930.8	C17:0, C24:0, C16:1	Heptadecanoic acid, lignoceric acid, palmitoleic acid
		0.1	C48:1	804.8	C18:0, C16:1, C14:0	Stearic acid, palmitoleic acid, myristic acid
		0.09	C54:5	880.8	C18:1, C18:1, C18:3	Oleic acid, oleic acid, linolenic acid
TAG	0.5	0.12	C52:1	860.8	C16:0, C18:1, C18:0	Palmitic acid, oleic acid, stearic acid
		0.11	C54:1	888.8	C18:0, C18:0, C18:1	Stearic acid, stearic acid, oleic acid
TC	0.4	0.35	C40:2	690.7	C6:0, C16:0, C18:2	Caproic acid, palmitic acid, linoleic acid
IL-6	0.6	0.13	C38:1	664.7	C10:0, C12:0, C16:1	Capric acid, lauric acid, palmitoleic acid
		0.1	C42:1	720.7	C16:0, C16:1, C10:0	Palmitic acid, palmitoleic acid, capric acid
		0.07	C56:1	916.8	C18:0, C18:0, C20:1	Stearic acid, stearic acid, gadoleic
HOMA-IR	0.5	0.09	C44:2	746.7	C18:2, C14:0, C12:0	Linoleic acid, myristic acid, lauric acid
		0.09	C56:7	904.8	C20:4, C18:1, C18:2	Arachidonic acid, oleic acid, linoleic acid
SC adipogenic	0.9	0.16	C58:10	926.8	C18:2, C18:2, C22:6	Linoleic acid, linoleic acid, docosahexaenoic acid
		0.16	C56:4	910.8	C18:1, C18:2, C20:1	Oleic acid, linoleic acid, gadoleic acid
		0.14	C57:4	924.7	C22:0, C19:4, C16:0	Behenic acid, C19:4, palmitic acid
		0.09	C40:1	692.7	C18:1, C16:0, C6:0	Oleic acid, palmitic acid, caproic acid
		0.08	C60:1	970.8	C24:0, C24:0, C18:1	Lignoceric acid, lignocerric acid, oleic acid
		0.22	C38:1	664.7	C18:1, C16:0, C4:0	Oleic acid, palmitic acid, butyric acid
OM adipogenic	1	0.18	C48:1	804.8	C18:0, C16:1, C14:0	Stearic acid, palmitoleic acid, myristic acid
		0.14	C49:1	818.7	C18:1, C17:0, C14:0	Oleic acid, heptadecanoic acid, myristic acid
		0.11	C56:1	916.8	C18:0, C18:0, C20:1	Stearic acid, stearic acid, gadoleic
		0.09	C54:0	890.8	C18:0, C18:0, C18:0	Stearic acid, stearic acid, stearic acid,
		0.06	C38:0	666.7	C10:0, C14:0, C14:0	Capric acid, myristic acid, myristic acid
		0.05	C56:2	914.8	C18:1, C18:1, C20:0	Oleic acid, oleic acid, arachidic acid
		0.04	C51:1	846.7	C18:1, C15:0, C18:0	Oleic acid, pentadecanoic acid, stearic acid

A step-wise regression was performed to identify the best TAG predictors of various traits. A p value significance level of 0.001 was used

## Discussion

TAGs constitute over 99% of lipid species in the adipose tissue of healthy individuals, with cholesterol and phospholipids making minor contributions [16]. TAGs are located within dynamic functional organelles known as lipid droplets that play important roles in intracellular vesicle trafficking, cell signaling and lipid homeostasis [17]. Although TAGs are not signaling molecules, fatty acids produced during their synthesis or breakdown were shown to interfere with the intracellular insulin signaling pathway and contribute to the development of insulin resistance [10]. Previous studies investigating TAG and fatty acid composition between subcutaneous and omental depots were published [6, 7]. However, this is the first study comparing TAGs and their fatty acid species in adipose tissues derived from IS, IR and T2DM obese individuals. Current technologies enable high-throughput profiling of the lipidome [18, 19]. In this study, LC/MS-based lipid profiling was performed to identify adipose signature of obesity-associated insulin sensitivity, insulin resistant and T2DM. The emerging data reveal differences in TAG species between SC and OM adipose tissues such as C38:1, C53:5, C51:3, C50:4, C59:1, C54:6 and C50:2 and among IS, IR and T2DM obese individuals including C46:4, C48:5, C48:4, C38:1, C50:3, C40:2, C56:3, C56:4, C56:7 and C58:7. The data also show differences in fatty acid compositions of TAGs associated with T2DM such as C12:0 and C18:3, suggesting a potential functional role of the identified species. Significant associations between the identified TAG species and traits of metabolic syndrome such as age, BMI, lipids (total cholesterol and circulating TAG), the inflammatory marker IL-6 and adipogenic capacity of preadipocytes derived from the same adipose tissues were identified. These associations could shed light on the molecular mechanisms contributing to the increased risk of metabolic disease.

### Depot specific differences

Our data identified few TAGs that were differently expressed between SC and OM tissues. One TAG that was higher in SC compared to OM was C38:1, which contains C4:0 (butyric acid). The latter was shown before to inhibit lipolysis and increase insulin sensitivity in primary rat adipocytes [20], perhaps contributing to the greater association of insulin resistance with OM mass compared to SC mass [21]. A previous study in obese men has shown increased C50:0, C59:2, C58:2, C60:3, C64:4, C51:0 and C65:1 fatty acids in OM compared to SC adipose tissues [6]. Changes in lipid composition between the two depots were attributed to differences in adipocyte differentiation, metabolism of the lipid droplet, and extent of beta-oxidation [6]. Differences between the two studies may reflect ethnic and/or diet differences between our Asian and the other study’s Caucasian population. Variations in fatty acid composition between SC and OM fat depots confirm the specific metabolism of each depot, as selective lipolytic and lipogenic mechanisms may function in each tissue depot. Indeed, studies have shown that desaturase enzymes, regulating the number of saturated fatty acids, exhibit a depot-specific profile [22] in close association with insulin resistance [23].

### IS, IR and T2DM specific differences

Systemic levels of fatty acid increase with obesity and T2DM, perhaps as a result of insulin resistance of adipose tissue and subsequent increased lipolysis; although in some obese individuals, fatty acid release from adipose tissues is reduced per kg fat in order to normalize plasma non-esterified fatty acid concentrations [24]. In our study, significant differences in levels of TAG composition were detected between IS, IR and T2DM. A number of candidates were either increased or decreased with risk of insulin resistance and T2DM, despite the predominant view of TAGs as an adverse risk factor for diabetes. Using the Wilcoxon sum of ranks statistics, fatty acids frequently occurring in highly-ranked TAGs along the list of TAGs ordered by p value from diabetes association analysis were revealed. Two fatty acids were identified: C12:0 and C18:3. The strength of this enrichment analysis approach is that, unlike the Fishers’ exact test, no arbitrary significance cut-off is applied on the list of TAGs. However, a possible weakness relates to the fact that since the TAGs are ordered by p value, no account is given to the direction of change and therefore one may not speak of depletion or increase in fatty acid TAG level but rather a dynamic in metabolic activity involving the fatty acid in association with the phenotype of interest. This was observed with C18:3, and a negative correlation was noted between C18:3 host TAGs found increased and others decreased with diabetes, effectively suggesting a metabolic link between the two sets of TAGs. Our findings confirm previous studies that showed significant correlations of specific fatty acids with insulin sensitivity. These include a cross-sectional analysis of adipose tissue biopsies from elderly obese men, which identified positive correlations between levels of C12:0, C18:2 and C18:3 and insulin sensitivity [25]. Our data also confirmed the association of C18:3 with metabolic status as shown previously in two groups of obese individuals who underwent weight loss surgery [26]. Furthermore, subjects in the most insulin-sensitive quintile showed a significantly higher percentage of circulating C18:2 (pre-cursor of C18:3) than the remaining subjects [27], further confirming our data. Functionally, previous work implicated C18:2 in the modulation of insulin signaling in rat skeletal muscle [28]. Therefore, our findings confirm previous results with regard to the association of C12:0 with insulin sensitivity [25], perhaps through triggering Glut4 translocation [27]. Our data also revealed reduction in C18:3 with T2DM incidents. This also confirms previous findings showing a negative correlation of C18:3 and its precursor with insulin resistance and positive association with insulin sensitivity [27].

### Association of TAGs with mediators of metabolic syndrome

Further, our data highlight a panel of TAGs that were associated with mediators of metabolic disease in obese individuals. Increased age was associated with accumulation of C56:1 that is composed of saturated fatty acids C20:0 and C18:0 and mono-unsaturated C18:1, whereas age was negatively correlated with C54:8 that is composed of unsaturated fatty acids C18:2 and C18:3. Although participants had comparable BMI, the small increase in BMI was positively correlated with three unsaturated TAGs (C57:1, C48:1 and C54:5). Whereas circulating TAGs were associated with accumulation of C52:1 and C54:1 in the adipose tissue, total cholesterol was positively correlated with C40:2. The negative correlation between IL-6 and C38:1, C42:1 and C56:1 may suggest an anti-inflammatory effect of fatty acids that constitute these TAGs, in particular C10:0 that was shown previously to exert an anti-inflammatory properties [29]. HOMA-IR was also negatively correlated with C44:2 and C56:7, both containing C18:2 shown previously to negatively correlate with insulin resistance [25].

### Association of TAGs with adipogenic capacity

Several TAGs were highly correlated with SC or OM adipogenic capacity. Previous studies have shown that the greater adipogenic capacity of SC and OM preadipocytes taken from IS obese individuals compared to IR and T2DM counterparts is partially mediated by lower IL-6 secretion and oxidative stress [12, 15, 30]. Secretion of interleukin IL-6 is significantly decreased after treatment with C18:2, C22:6 and C16:0 via inhibition of nuclear factor kappa B (NF-κB) and subsequent activation of the master regulator of adipogenesis, PPARγ [31]. Our data revealed positive correlations of C56:4 and C57:4, containing C18:2, C16:0, with SC adipogenic capacity. OM adipogenic capacity was associated with C49:1, C38:0 and C56:2, containing C16:0, C18:1 and C14:0. These fatty acids were shown previously to induce adipocyte differentiation in rodents [32–36] and potentially play a similar role in human preadipocytes.

### Study limitations

One main limitation of this study is the relatively low number of participants, especially in the T2DM group. Additionally, the difference in gender distribution between IS and IR groups (predominantly females) and T2DM (predominantly males) group may have introduced bias in the study design that may have influenced the results. Despite these factors, clear TAG and fatty acid signatures were identified after correcting for potential confounders such as gender and BMI. Another limitation of the current work is its focus on association of TAGs with insulin resistance and risk of T2DM without an absolute quantitation of any specific analyte. Incorporating isotope-labeled standards would allow absolute quantitation and improve the precision of measurements. Finally, differences in TAG composition in adipose tissues among the studied groups may have been influenced by their diet. Indeed, previous studies have shown that the process of fatty acid and TAG deposition in rat adipose tissue depends on the composition of the diet [37]. Dietary linoleic acid content was shown to influence the distribution of TAG species in rat adipose tissue, particularly di- and trilinoleoyl containing TAG as a result of linoleic acid intake [38]. Other studies have shown that the composition of TAG in rat epididymal, subcutaneous and perirenal adipose tissues was broadly reflecting dietary oils such as isomeric octadecenoic acids from coriander oil and high oleic sunflower oil [39]. Taken all these limitations into account, confirmation in different populations is warranted to validate these findings.

## Conclusion

In summary, our data supports the dynamic nature of adipose tissue and the complex interaction between adipose tissue physiology and its lipid composition. The TAGs and their fatty acid composition within human adipose tissues from obese subjects are markedly different, depending on the insulin sensitivity status of the donors. Our data suggest that adipose tissues from obese IR and T2DM individuals exhibit TAG-specific signatures that may contribute to their increased risk compared to their insulin-sensitive counterparts or could reflect different dietary consumption among the studied groups. Future experiments are warranted to investigate the functional relevance of these specific lipidomic profiles with reference to participants’ consumed diet.

### Additional files

**Additional file 1: Table S1.** Comparison of participants’ characteristics by gender. **Table S2.** Differences between obese and morbidly obese subjects. **Table S3.** TAGs exhibiting BMI interaction.

**Additional file 2: Figure S1.** Adipogenic capacity of preadipocytes derived from subcutaneous (SC) and omental (OM) adipose tissues from insulin sensitive (IS), insulin resistant (IR) and type 2 diabetes mellitus (T2DM) patients. Representative images of SC and OM adipocytes form IS and IR individuals stained with DAPI in blue (nuclear staining) and lipidtox in green (lipid droplet staining) (A). A bar chart showing differences in the adipogenic capacity (percentage of differentiated adipocytes to total number of nuclei) in SC and OM preadipocytes derived from IS, IR and T2DM individuals (B). Significant differences in adipogenic capacity with disease progression were detected as reported previously [12, 15].

## Appendix

See Table 6.

**Table 6:** List of identified TAG, their molecular weights (MW), fatty acid compositions and fatty acids identities

Component name	TAG	MW	Fatty acid composition	Fatty acids identities
TAG2	C38:1	664.7	C10:0, C12:0, C16:1	Capric acid, lauric acid, palmitoleic acid
TAG2A	C38:1	664.7	C18:1, C16:0, C4:0	Oleic acid, palmitic acid, butyric acid
TAG3	C38:0	666.7	C12:0, C12:0, C14:0	Lauric acid, lauric acid, myristic acid
TAG7	C40:2	690.7	C6:0, C16:0, C18:2	Caproic acid, palmitic acid, linoleic acid
TAG84	C40:1	692.7	C18:1, C16:0, C6:0	Oleic acid, palmitic acid, caproic acid
TAG9	C42:2	718.7	C18:2, C12:0, C12:0	Linoleic acid, lauric acid, lauric acid
TAG10	C42:1	720.7	C12:0, C14:0, C16:1	Lauric acid, myristic acid, palmitoleic acid
TAG12	C44:3	744.7	C18:2, C16:1, C10:0	Linoleic acid, palmitoleic acid, capric acid
TAG13	C44:2	746.7	C18:2, C14:0, C12:0	Linoleic acid, myristic acid, lauric acid
TAG14	C44:1	748.7	C18:1, C16:0, C10:0	Oleic acid, palmitic acid, capric acid
TAG16A	C46:4	770.7	C18:2, C18:2, C10:0	Linoleic acid, linoleic acid, capric acid
TAG17	C46:3	772.7	C18:2, C16:1, C12:0	Linoleic acid, palmitoleic acid, lauric acid
TAG18	C46:2	774.8	C18:2, C16:0, C12:0	Linoleic acid, palmitic acid, lauric acid
TAG19	C46:1	776.8	C18:1, C16:0, C12:0	Oleic acid, palmitic acid, lauric acid
TAG20	C46:0	778.8	C16:0, C16:0, C14:0	Palmitic acid, palmitic acid, myristic acid
TAG21	C48:5	796.7	C18:2, C18:3, C12:0	Linoleic acid, linolenic acid, lauric acid
TAG22	C48:4	798.7	C18:2, C18:2, C12:0	Linoleic acid, linoleic acid, lauric acid
TAG23	C48:3	800.8	C18:2, C16:1, C14:0	Linoleic acid, palmitoleic acid, myristic acid
TAG24	C48:2	802.8	C18:2, C16:0, C14:0	Linoleic acid, palmitic acid, myristic acid
TAG24A	C48:2	802.8	C18:1, C16:1, C14:0	Oleic acid, palmitoleic acid, myristic acid
TAG25	C48:1	804.8	C18:1, C16:0, C14:0	Oleic acid, palmitic acid, myristic acid
TAG26	C48:0	806.7	C16:0, C16:0, C16:0	Palmitic acid, palmitic acid, palmitic acid
TAG27	C48:	818.7	C18:1, C16:0, C14:	Oleic acid, palmitic acid, pentadecylic acid
TAG29	C50:6	822.7	C18:3, C18:3, C14:0	Linolenic acid, linolenic acid, myristic acid
TAG30	C50:5	824.7	C18:3, C16:1, C16:1	Linolenic acid, palmitoleic acid, palmitoleic acid
TAG31	C50:4	826.7	C18:2, C18:2, C14:0	Linoleic acid, linoleic acid, myristic acid
TAG32	C50:3	828.8	C16:1, C16:1, C18:1	Palmitoleic acid, palmitoleic acid, oleic acid
TAG33	C50:2	830.8	C18:2, C16:0, C16:0	Linoleic acid, palmitic acid, palmitic acid
TAG34	C50:1	832.8	C18:1, C16:0, C16:0	Oleic acid, palmitic acid, palmitic acid
TAG35	C50:0	834.7	C18:0, C16:0, C16:0	Stearic acid, palmitic acid, palmitic acid
TAG36	C51:3	842.6	C18:1, C16:1, C17:1	Oleic acid, palmitoleic acid, *cis*-10-heptadecenoic acid
TAG37	C50:	844.7	C18:1, C18:1, C14:	Oleic acid, oleic acid, pentadecylic acid
TAG38	C51:1	846.7	C18:1, C17:0, C16:0	Oleic acid, heptadecanoic acid, palmitic acid
TAG39	C52:7	848.7	C18:3, C18:3, C16:1	Linolenic acid, linolenic acid, palmitoleic acid
TAG39A	C51:0	848.7	C18:0, C17:0, C16:0	Stearic acid, heptadecanoic acid, palmitic acid
TAG40	C52:6	850.7	C18:2, C14:0, C20:4	Linoleic acid, myristic acid, C20:4
TAG40A	C52:6	850.7	C18:3, C16:0, C18:3	Linolenic acid, palmitic acid, linolenic acid
TAG41	C52:5	852.7	C18:2, C18:2, C16:1	Linoleic acid, linoleic acid, palmitoleic acid
TAG42	C52:4	854.8	C18:2, C18:2, C16:0	Linoleic acid, linoleic acid, palmitic acid
TAG43	C52:3	856.8	C18:1, C18:1, C16:1	Oleic acid, oleic acid, palmitoleic acid
TAG44	C52:2	858.8	C18:1, C18:1, C16:0	Oleic acid, oleic acid, palmitic acid
TAG45	C52:1	860.8	C16:0, C18:1, C18:0	Palmitic acid, oleic acid, stearic acid
TAG46	C52:0	862.7	C18:0, C18:0, C16:0	Stearic acid, stearic acid, palmitic acid
TAG47	C53:5	866.7	C17:0, C17:1, C19:4	Heptadecanoic acid, *cis*-10-heptadecanoic acid, C19:4
TAG49A	C53:1	870.7	C18:1, C18:2, C17:0	Oleic acid, linoleic acid, heptadecanoic acid
TAG52	C54:7	876.7	C18:2, C18:2, C18:3	Linoleic acid, linoleic acid, linolenic acid
TAG52A	C54:7	876.7	C18:2, C18:2, C18:3	Linoleic acid, linoleic acid, linolenic acid
TAG53	C54:6	878.7	C18:2, C18:1, C18:3	Linoleic acid, oleic acid, linolenic acid
TAG53A	C54:6	878.7	C18:2, C18:2, C18:2	Linoleic acid, linoleic acid, linoleic acid
TAG54	C54:5	880.8	C18:2, C18:2, C18:1	Linoleic acid, linoleic acid, oleic acid
TAG54A	C54:5	880.8	C18:2, C18:2, C18:1	Linoleic acid, linoleic acid, oleic acid
TAG55	C54:4	882.8	C18:1, C18:1, C18:2	Oleic acid, oleic acid, linoleic acid
TAG56A	C54:3	884.8	C18:1, C18:1, C18:1	Oleic acid, oleic acid, oleic acid
TAG57	C54:2	886.8	C18:1, C18:1, C18:0	Oleic acid, oleic acid, stearic acid
TAG58A	C54:1	888.8	C18:0, C18:0, C18:1	Stearic acid, stearic acid, oleic acid
TAG59	C54:0	890.8	C18:0, C18:0, C18:0	Stearic acid, stearic acid, stearic acid
TAG60	C56:8	902.8	C20:5, C18:1, C18:2	Eicosapentaenoic acid, oleic acid, linoleic acid
TAG60A	C56:8	902.8	C20:5, C18:1, C18:2	Eicosapentaenoic acid, oleic acid, linoleic acid
TAG60B	C55:1	902.8	C20:0, C18:1, C17:0	Eicosapentaenoic acid, oleic acid, linoleic acid
TAG61	C56:7	904.8	C20:4, C18:1, C18:2	Arachidonic acid, oleic acid, linoleic acid
TAG64A	C56:4	910.8	C18:1, C18:2, C20:1	Oleic acid, linoleic acid, gadoleic
TAG65	C56:3	912.8	C20:1, C18:1, C18:1	Gadoleic, oleic acid, oleic acid
TAG66	C56:2	914.8	C18:1, C18:1, C20:0	Oleic acid, oleic acid, arachidic acid
TAG67	C56:1	916.8	C20:0, C18:0, C18:1	Arachidic acid, stearic acid, oleic acid
TAG67A	C56:1	916.8	C18:0, C18:0, C20:1	Stearic acid, stearic acid, gadoleic
TAG71	C58:9	928.8	C22:6, C18:1, C18:2	Docosahexaenoic acid, oleic acid, linoleic acid
TAG71A	C58:9	928.8	C22:6, C18:1, C18:2	Docosahexaenoic acid, Oleic acid, linoleic acid
TAG71B	C58:9	928.8	C22:6, C18:1, C18:2	Docosahexaenoic acid, oleic acid, linoleic acid
TAG74	C58:7	934.8	C18:6, C24:0, C16:1	C18:6, lignoceric acid, palmitoleic acid
TAG75	C58:4	938.7	C18:3, C24:0, C16:1	Linolenic acid, lignoceric acid, palmitic acid
TAG78	C58:2	942.8	C20:1, C20:1, C18:0	Gadoleic, gadoleic, stearic acid
TAG79A	C58:1	944.8	C22:0, C18:0, C18:1	Behenic acid, stearic acid, oleic acid
TAG80	C64:1	956.8	C23:0, C23:0, C18:1	Tricosanoic acid, tricosanoic acid, oleic acid,
TAG81	C59:1	958.8	C23:0, C18:0, C18:1	Tricosanoic acid, stearic acid, oleic acid
TAG82	C60:3	968.8	C24:0, C18:1, C18:2	Lignocerric acid, oleic acid, linoleic acid
TAG83	C60:1	970.8	C24:0, C24:0, C18:1	Lignocerric acid, lignocerric acid, oleic acid

## Abbreviations

DAPI: 4′,6-diamidino-2-phenylindole
BMI: body mass index
DBP: diastolic blood pressure
DAGs: diacylglycerols
ECN: effective carbon number
FPG: fasting blood glucose
HDL: high density lipoprotein
HOMA-IR: homeostatic model assessment
IS: insulin sensitive
IR: insulin resistant
IL-6: interleukin 6
LDL: low density lipoprotein
MAP: mean arterial blood pressure
NARP: non-aqueous reverse phase UHPLC separation
NF-κB: nuclear factor kappa B
OM: omental
OPLS-DA: orthogonal partial least square discriminate analysis
PCA: principle component analysis
RT: retention time
SBP: systolic blood pressure
SVF: stromal vascular fraction
SC: subcutaneous
TAGs: triacylglycerols
T2DM: type 2 diabetes mellitus
UHPLC: ultra-high performance liquid chromatography
