# Supplementary material for: Triglyceride profiling in adipose tissues from obese insulin sensitive, insulin resistant and type 2 diabetes mellitus individuals
Source: J Transl Med. 2018 Jun 26;16:175. doi: 10.1186/s12967-018-1548-x (PMC6019324; doi:10.1186/s12967-018-1548-x)
Supplement: Supplementary file 1 — Additional file 1: Table S1. Comparison of participants’ characteristics by gender. Table S2. Differences between obese and morbidly obese subjects. Table S3. TAGs exhibiting BMI interaction. [file 12967_2018_1548_MOESM1_ESM.docx]

**Additional file**

**Table S1.** Comparison of participants’ characteristics by gender.

|  |  | **All (44F, 20M)** | | | **IS (14F, 4M)** | | | **IR (24F, 11M)** | | | **T2DM (6F, 5M)** | | |
| --- | --- | --- | --- | --- | --- | --- | --- | --- | --- | --- | --- | --- | --- |
| **Variables** | **Gender** | **Mean** | **SD** | **P value** | **Mean** | **SD** | **P value** | **Mean** | **SD** | **P value** | **Mean** | **SD** | **P value** |
| **Age (year)** | Female | 31.5 | 9.3 | 0.408 | 32.4 | 8.2 | 0.780 | 29.5 | 9 | 0.430 | 41 | 12.2 | 0.450 |
|  | Male | 33.6 | 11.6 |  | 31 | 15.3 |  | 32 | 10.1 |  | 47 | 2.6 |  |
| **BMI (kg.m-2)** | Female | 42.1 | 7.2 | 0.110 | 41.5 | 7.8 | 0.920 | 41.5 | 5.9 | 0.010 | 49.4 | 11.3 | 0.260 |
|  | Male | 45.0 | 6.7 |  | 41.1 | 3.6 |  | 47.1 | 7.3 |  | 40.4 | 4.4 |  |
| **SBP (mmHg)** | Female | 119.8 | 13.9 | 0.008 | 119 | 15 | 0.980 | 117.8 | 13.3 | 0.000 | 133.5 | 11.3 | 0.750 |
|  | Male | 129.6 | 15.0 |  | 118.8 | 10.3 |  | 132.8 | 16.1 |  | 130.3 | 14.2 |  |
| **DBP (mmHg)** | Female | 66.6 | 9.1 | 0.001 | 66.6 | 9.6 | 0.670 | 64.6 | 8.1 | 0.000 | 73.8 | 8.2 | 0.860 |
|  | Male | 76.0 | 13.8 |  | 64.4 | 11.1 |  | 80.6 | 13.7 |  | 72.5 | 6.4 |  |
| **MAP** | Female | 84.7 | 8.8 | 0.001 | 83.7 | 7.5 | 0.780 | 82.7 | 8.2 | 0.000 | 95.2 | 9.5 | 0.570 |
|  | Male | 93.7 | 13.1 |  | 82.5 | 10.6 |  | 98 | 12.8 |  | 90.2 | 9.7 |  |
| **Cholesterol (mmol/L)** | Female | 4.6 | 1.2 | 0.466 | 4.4 | 1 | 0.710 | 4.5 | 1.4 | 0.470 | 5 | 1.1 | 0.460 |
|  | Male | 4.8 | 0.8 |  | 4.3 | 0.5 |  | 4.8 | 0.9 |  | 5.5 | 0.4 |  |
| **LDL (mmol/L)** | Female | 2.8 | 0.8 | 0.169 | 2.7 | 0.9 | 0.880 | 2.8 | 0.8 | 0.190 | 2.9 | 1.3 | 0.590 |
|  | Male | 3.1 | 0.8 |  | 2.7 | 0.3 |  | 3.2 | 0.9 |  | 3.4 | 0.8 |  |
| **HDL (mmol/L)** | Female | 1.5 | 0.8 | 0.048 | 1.4 | 0.3 | 0.310 | 1.6 | 1.1 | 0.100 | 1.4 | 0.2 | 0.280 |
|  | Male | 1.1 | 0.3 |  | 1.2 | 0.4 |  | 1.1 | 0.3 |  | 1.2 | 0.3 |  |
| **Triglyceride (mmol/L)** | Female | 1.3 | 1.0 | 0.490 | 1.2 | 0.7 | 0.480 | 1.2 | 0.6 | 0.170 | 1.5 | 0.6 | 0.330 |
|  | Male | 1.5 | 0.8 |  | 1 | 0.3 |  | 1.5 | 0.7 |  | 2.4 | 1.5 |  |
| **Leptin (ng/ml)** | Female | 67.3 | 24.1 | 0.000 | 70.1 | 25.2 | 0.040 | 67.4 | 22.1 | 0.010 | 55.6 | 33.4 | 0.110 |
|  | Male | 42.0 | 16.9 |  | 41.5 | 8 |  | 48.4 | 16.1 |  | 17.2 | 5.6 |  |
| **IL-6 (pg/ml)** | Female | 3.7 | 2.0 | 0.594 | 3.5 | 1.9 | 0.260 | 3.6 | 1.9 | 0.690 | 4.7 | 2.7 | 0.400 |
|  | Male | 3.4 | 1.5 |  | 2.3 | 0.9 |  | 3.9 | 1.7 |  | 3.2 | 0.3 |  |
| **FBG (mmol/L)** | Female | 9.9 | 6.5 | 0.259 | 6.3 | 2.2 | 0.050 | 11.3 | 7.3 | 0.110 | 15.5 | 6 | 0.170 |
|  | Male | 12.1 | 9.8 |  | 3.8 | 2.8 |  | 15.7 | 10 |  | 7.4 | 4.2 |  |
| **Insulin (mIU/L)** | Female | 9.9 | 6.7 | 0.554 | 6.7 | 1.9 | 0.080 | 12.2 | 8.2 | 0.750 | 10 | 4.4 | 0.410 |
|  | Male | 11.2 | 11.2 |  | 5 | 1.2 |  | 13.3 | 13.4 |  | 14.5 | 8.7 |  |
| **HOMA-IR** | Female | 3.6 | 2.1 | 0.115 | 1.8 | 0.4 | 0.000 | 4.4 | 1.6 | 0.040 | 6.5 | 2.8 | 0.930 |
|  | Male | 4.7 | 3.3 |  | 0.8 | 0.7 |  | 5.8 | 2.5 |  | 6.8 | 5.4 |  |

**Table S2.** Differences between obese and morbidly obese subjects.

| Obese (n=26),  Morbidly Obese (n=40) | | Mean | Std. Deviation | P value |
| --- | --- | --- | --- | --- |
| Age | Obese | 30.3 | 9.1 | 0.166 |
|  | Morbidly obese | 33.7 | 10.4 |  |
| BMI | Obese | 36.8 | 2.8 | 0.000 |
|  | Morbidly obese | 46.8 | 6.2 |  |
| SBP | Obese | 117.9 | 13.7 | 0.033 |
|  | Morbidly obese | 125.6 | 15.1 |  |
| DBP | Obese | 67.2 | 5.8 | 0.260 |
|  | Morbidly obese | 70.5 | 13.6 |  |
| MAP | Obese | 84.2 | 7.0 | 0.071 |
|  | Morbidly obese | 89.3 | 12.6 |  |
| Cholesterol | Obese | 4.7 | 1.1 | 0.675 |
|  | Morbidly obese | 4.6 | 1.2 |  |
| LDL | Obese | 2.9 | 0.7 | 0.856 |
|  | Morbidly obese | 2.9 | 0.9 |  |
| HDLC | Obese | 1.3 | 0.3 | 0.758 |
|  | Morbidly obese | 1.4 | 0.9 |  |
| Triglyceride | Obese | 1.2 | 0.6 | 0.118 |
|  | Morbidly obese | 1.5 | 1.1 |  |
| Leptin | Obese | 58.9 | 27.2 | 0.899 |
|  | Morbidly obese | 59.7 | 24.0 |  |
| Adiponectin | Obese | 3.8 | 2.2 | 0.393 |
|  | Morbidly obese | 3.2 | 1.6 |  |
| IL6 | Obese | 2.7 | 1.6 | 0.005 |
|  | Morbidly obese | 4.1 | 1.8 |  |
| FBG | Obese | 7.9 | 5.7 | 0.025 |
|  | Morbidly obese | 12.1 | 8.3 |  |
| Insulin | Obese | 10.5 | 6.0 | 0.911 |
|  | Morbidly obese | 10.3 | 9.6 |  |
| HOMA | Obese | 3.1 | 1.6 | 0.034 |
|  | Morbidly obese | 4.5 | 2.9 |  |

**Table S3.** TAGs exhibiting BMI interaction.

| ID | TAG | Fatty Acid Composition | Fatty Acids Identities | BMI | Fold Change | Std.Error | p-value | FDR  p-value |
| --- | --- | --- | --- | --- | --- | --- | --- | --- |
| TAG48 | C53:4 | Stearic acid, Linolenic acid, Cis-10-Heptadecanoic acid | IS vs T2DM | low | 0.78 | 0.20 | 0.0003 | 0.04 |
|  |  |  | IS vs IR | low | 0.12 | 0.12 | 0.35 | 0.71 |
|  |  |  | IS vs T2DM | high | 0.16 | 0.18 | 0.36 | 0.73 |
|  |  |  | IS vs IR | high | 0.01 | 0.10 | 0.91 | 0.98 |
| TAG7 | C40:2 | Caproic acid ,palmitic acid, linoleic acid | IS vs T2DM | low | -1.61 | 0.32 | 4.42167E-06 | 0.002 |
|  |  |  | IS vs IR | low | -0.58 | 0.21 | 0.008 | 0.16 |
|  |  |  | IS vs T2DM | high | -0.69 | 0.29 | 0.02 | 0.25 |
|  |  |  | IS vs IR | high | -0.03 | 0.17 | 0.82 | 0.95 |
