# Supplementary figures and images for: Triglyceride profiling in adipose tissues from obese insulin sensitive, insulin resistant and type 2 diabetes mellitus individuals
Source: J Transl Med. 2018 Jun 26;16:175. doi: 10.1186/s12967-018-1548-x (PMC6019324; doi:10.1186/s12967-018-1548-x)

Additional file 2: Figure S1.


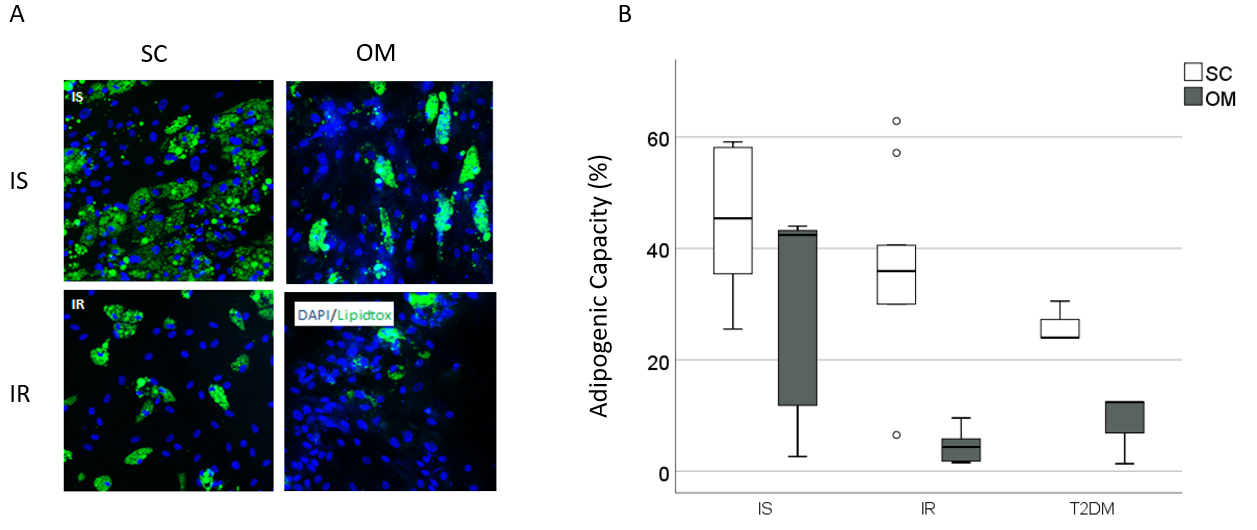

Supplement: Supplementary file 2 — Additional file 2: Figure S1. Adipogenic capacity of preadipocytes derived from subcutaneous (SC) and omental (OM) adipose tissues from insulin sensitive (IS), insulin resistant (IR) and type 2 diabetes mellitus (T2DM) patients. Representative images of SC and OM adipocytes form IS and IR individuals stained with DAPI in blue (nuclear staining) and lipidtox in green (lipid droplet staining) (A). A bar chart showing differences in the adipogenic capacity (percentage of differentiated adipocytes to total number of nuclei) in SC and OM preadipocytes derived from IS, IR and T2DM individuals (B). Significant differences in adipogenic capacity with disease progression were detected as reported previously [12, 15]. [file 12967_2018_1548_MOESM2_ESM.docx]
